# Discovery and development of the Polo-like kinase inhibitor volasertib in cancer therapy

**DOI:** 10.1038/leu.2014.222

**Published:** 2014-08-15

**Authors:** B T Gjertsen, P Schöffski

**Affiliations:** 1 Centre for Cancer Biomarkers (CCBIO), Department of Clinical Science, University of Bergen, Bergen, Norway; 2 Department of Internal Medicine, Haematology Section, Haukeland University Hospital, Bergen, Norway; 3 Department of General Medical Oncology, Leuven Cancer Institute, University Hospitals Leuven, Katholieke Universiteit Leuven, Leuven, Belgium

## Abstract

Owing to their integral involvement in cell cycle regulation, the Polo-like kinase (Plk) family, particularly Plk1, has emerged as an attractive therapeutic target in oncology. In recent years, several Plk1 inhibitors have been developed, with some agents showing encouraging results in early-phase clinical trials. This review focuses on volasertib (BI 6727; an investigational agent), a potent and selective Plk inhibitor. Volasertib has shown promising activity in various cancer cell lines and xenograft models of human cancer. Trials performed to date suggest that volasertib has clinical efficacy in a range of malignancies, with the most promising results seen in patients with acute myeloid leukemia (AML). Encouragingly, recent phase II data have demonstrated that volasertib combined with low-dose cytarabine (LDAC) was associated with higher response rates and improved event-free survival than LDAC alone in patients with previously untreated AML. Based on these observations, and its presumably manageable safety profile, volasertib is currently in phase III development as a potential treatment for patients with AML who are ineligible for intensive remission induction therapy. Given that many patients with AML are of an older age and frail, this constitutes an area of major unmet need. In this review, we discuss the biologic rationale for Plk1 inhibitors in cancer, the clinical development of volasertib to date in solid tumors and AML, and the future identification of biomarkers that might predict response to volasertib and help determine the role of this agent in the clinic.

## Introduction

**Figure 1:**
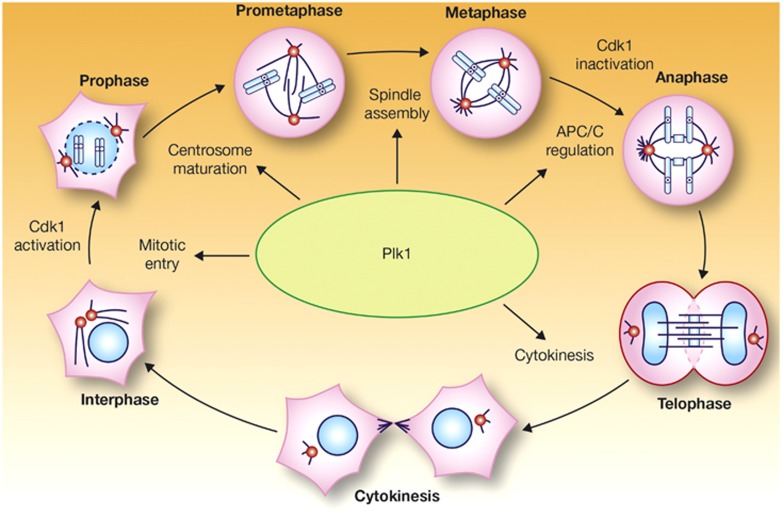
Functions of Plk1 during mitosis. APC/C, anaphase-promoting complex/cyclosome; Cdk1, cyclin-dependent kinase 1. Reprinted by permission from Macmillan Publishers Ltd: *Nature Reviews Molecular Cell Biology* (Barr *et al.*^1^), copyright 2004.

The Polo-like kinases (Plks) comprise a family of five serine/threonine protein kinases that have key roles in many processes involved in control of the cell cycle, including entry into mitosis, DNA replication and the stress response to DNA damage. However, Plk1 is deemed especially important and has been the focus of the majority of Plk research. Plk1, which is activated by another kinase, Aurora A, has multiple regulatory roles in the cell cycle, including the control of cell cycle progression into mitosis (Figure 1).^1,2^ Although the majority of studies highlight the role of Plk1 in mitosis, non-mitotic roles for Plk1 have also been suggested, including protection against apoptosis,^3,4^ and as a regulator of cancer cell invasiveness.^5^ Overexpression of Plk1 has been observed in a variety of solid tumors as well as in acute myeloid leukemia (AML),^6, 7, 8^ and has often been correlated with poor prognosis, disease stage, histologic grade, metastatic potential and survival.^9,10^ These observations have prompted research into the potential therapeutic application of Plk inhibitors in cancer.

One of the first Plk inhibitors to be developed was BI 2536. This selective, small-molecule, dihydropteridinone-derived, adenosine triphosphate-competitive kinase inhibitor of Plks 1–3 showed promise in preclinical experiments, validating Plks as potential drug targets in oncology.^7,11^ Unfortunately, phase II trials in patients with relapsed/refractory solid tumors,^12^ non-small-cell lung cancer (NSCLC),^13^ small-cell lung cancer,^14^ chemotherapy-naive pancreatic cancer^15^ and relapsed/refractory AML^16^ demonstrated that BI 2536 had modest, if any, clinical activity and was associated with a generally manageable safety profile. These disappointing results were thought to be attributable to the pharmacokinetic (PK) profile of BI 2536, particularly its relatively short terminal half-life (~50 h in patients with advanced solid tumors)^17^ and low intratumoral exposure (as observed in hepatocellular carcinoma samples derived from murine xenograft models), despite a large distribution volume.^18^ Clinical development of BI 2536 was halted and continued development of novel Plk1 inhibitors focused on improving PK profiles to facilitate sustained exposure to tumor tissues.

In this review, we focus on the discovery and development of volasertib, a selective and potent cell cycle kinase inhibitor that induces Polo arrest and apoptosis by targeting Plk1. Volasertib is currently the most clinically advanced of the investigational Plk inhibitors and this review discusses its development to date in solid tumors and AML. In addition, we look forward to the possible development of biomarkers of volasertib response that could help drive future treatment decisions in individual patients.

## Preclinical development of volasertib

**Figure 2:**
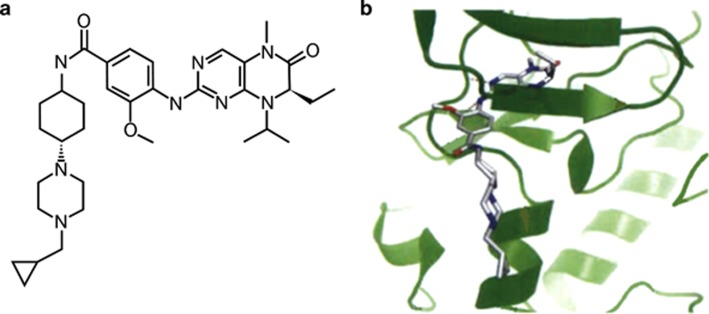
Chemical and X-ray structure of volasertib. Chemical structure (**a**) and binding mode (**b**) of volasertib in the adenosine triphosphate-binding pocket of human Plk1. Reprinted from *Clinical Cancer Research*, Copyright 2009, 15/9, 3094–3102, Rudolph *et al.*^19^ ‘BI 6727, a Polo-like kinase inhibitor with improved pharmacokinetic profile and broad antitumor activity', with permission from AACR.

Similar to BI 2536, a compound originally identified by screening a library of organic compounds for the capacity to inhibit the catalytic activity of Plk1,^11^ volasertib is a dihydropteridinone derivative and acts as a small-molecule, adenosine triphosphate-competitive kinase inhibitor of Plk1.^19^ Both agents are potent and selective inhibitors of Plk1; however, volasertib was derived by tailoring the dihydropteridinone structure of BI 2536 based on the criteria of potency, selectivity, ability to inhibit cell proliferation *in vitro*, efficacy in xenograft models of cancer, drug metabolism and PK characteristics.^19^ The chemical structure and binding mode of volasertib to the adenosine triphosphate-binding pocket of human Plk1 are shown in Figures 2a and b, respectively. It is important to note that volasertib is a unique chemical entity whose chemical structure and PK profile differ significantly compared with BI 2536. As a result, any insights regarding efficacy and safety previously determined with BI 2536 cannot be simply transferred to volasertib.

**Figure 3:**
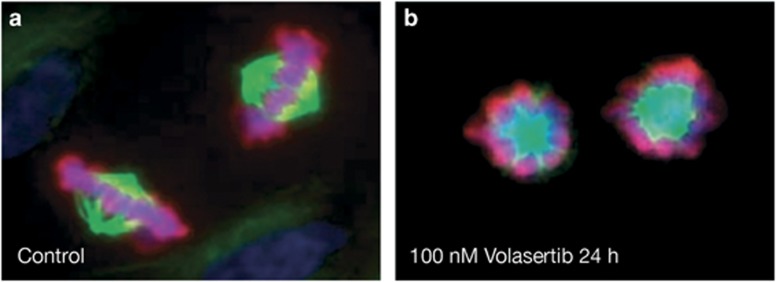
Volasertib prevents bipolar spindle formation, inducing cell cycle arrest in the early M phase. Immunofluorescence analysis of NCI-H460 NSCLC cells treated for 24 h with either (**a**) 0.1% dimethyl sulfoxide or (**b**) 100 nM/l volasertib. Cells were fixed and stained with 4', 6-diamidino-2-phenylindole (to stain DNA; blue), anti-tubulin (to stain spindles; green) and anti-phosphoSer10 histone H3 (pink). Volasertib treatment resulted in an accumulation of mitotic cells with monopolar spindles in which the kinetochores were not properly attached to the spindle—a cellular phenotype termed ‘Polo arrest'. Reprinted from *Clinical Cancer Research*, Copyright 2009, 15/9, 3094–3102, Rudolph *et al.*^19^ ‘BI 6727, a Polo-like kinase inhibitor with improved pharmacokinetic profile and broad antitumor activity', with permission from AACR.

Volasertib potently inhibits Plk1 (half-maximal inhibitory concentration (IC_50_) 0.87 nmol/l) as well as the two closely related kinases, Plk2 (IC_50_ 5  nmol/l) and Plk3 (IC_50_ 56 nmol/l), compared with IC_50_ values for BI 2536 of 0.83, 3.5 and 9.0 nmol/l, respectively,^11^ but shows no inhibitory activity against a panel of other unrelated kinases at concentrations up to 10 μmol/l (Table 1).^19^ Preclinical studies of volasertib in various tumor cell lines (colon, lung, melanoma, hematopoietic malignancies, prostate, urothelial and multiple pediatric tumors) have demonstrated inhibition of cell division that ultimately results in cell death.^19, 20, 21, 22, 23, 24, 25^ Targeting Plk with volasertib leads to a disruption of the mitotic spindle assembly resulting in a distinct mitotic arrest phenotype (‘Polo arrest') in prometaphase, accumulation of phospho-histone H3 and formation of aberrant monopolar mitotic spindles followed by apoptosis (Figure 3).^19,20,22,24,26^ Volasertib has also been shown to inhibit the activity of the bromodomain and extraterminal family protein BRD4 in biochemical assays *in vitro*.^27^ BRD4, as well as fms-like tyrosine kinase 3 (FLT3), have been suggested as drivers in the pathogenesis of AML,^28,29^ and preclinical studies of BRD4 inhibitors suggest the potential of these agents as antileukemic drugs.^30,31^ Although volasertib shows effects on BRD4,^27^ the cellular effects on BRD4 biomarker modulation are only observed at a concentration of 300 nM, suggesting that the effect on BRD4 may not be a relevant mechanism of action of volasertib.

Proteomic analyses of Plk-phosphorylated proteins using BI 2536 have identified hundreds of proteins normally affected by phosphorylation by Plk1, illustrating the importance of Plk1 in mitosis and other processes.^32^ Cellular proliferation is potently inhibited by volasertib, which acts on all proliferating cells, in a multitude of cancer cell lines, including colon (HCT116), lung (NCI-H460), melanoma (BRO), non-Hodgkin lymphoma (GRANTA-519, Raji) and AML (HL-60, THP-1) cells with half-maximal effective concentration values of 11–37 nmol/l.^19^ No correlation of Plk1 mRNA expression with sensitivity was observed in a data set based on 240 cancer cell lines derived from a variety of cancer indications treated with volasertib *in vitro* (unpublished data; Boehringer Ingelheim, Ingelheim, Germany). Volasertib also inhibited the growth and survival of cell lines derived from patients with pediatric acute lymphoblastic leukemia.^25^

**Figure 4:**
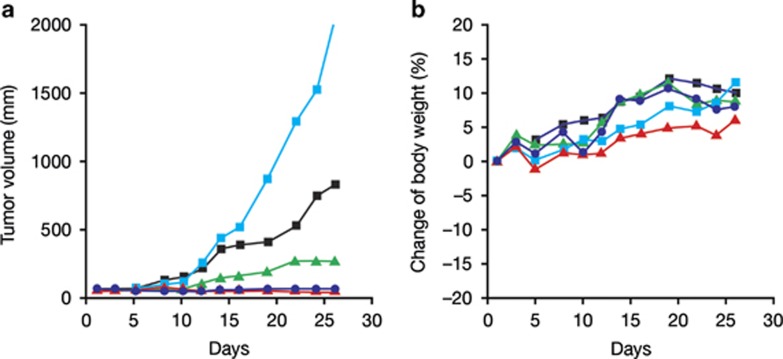
Efficacy and tolerability of volasertib in human AML xenograft model. Nude mice bearing established subcutaneous MV4-11 AML tumors with an average size of ~65 mm^3^ were treated intravenously for 4 weeks with either vehicle (light blue squares) or volasertib at 40 mg/kg (blue circles), 20 mg/kg (green triangles), or 10 mg/kg once a week (black squares), or at 20 mg/kg two times a week on consecutive days (red triangles). Median tumor volumes of eight animals per treatment group (**a**) and median body weight change as % of initial body weight (**b**) are shown. Efficacy has also been demonstrated in three disseminated AML models (MV4-11 (*FLT3*-ITD/*FLT3*-ITD), Molm-13 (*FLT3*-ITD/wild-type *FLT3*) and THP-1 (wild-type *FLT3*/wild-type *FLT3*) cell lines).

In colon (HCT116) and lung (NCI-H460) xenograft tumor models, volasertib monotherapy was associated with reduced tumor growth, including growth delays and tumor regressions.^19^ Consistent with the *in vitro* data, volasertib treatment led to cell cycle arrest and apoptosis in tumor samples derived from tumor-bearing mice.^19^ Volasertib concentrations measured in extracts from the tumors, multiple organs (brain, kidney, liver, lung and muscle) and plasma samples from these mice suggest good tissue penetration in all organs tested, although the central nervous system exposure is notably lower than the exposure observed for the other organs and does not exceed levels observed in the plasma.^19^ Marked antitumor activity and good tolerability were also observed in xenograft models of AML (Figure 4), human melanoma^33^ and various pediatric cancers.^23,24^ An improvement in antitumor control was observed with volasertib plus whole-body irradiation in a xenograft model of squamous cell carcinoma, likely as a result of concomitant cell cycle inhibition and cytotoxic effects of this combination.^34^ Preclinical PK data showed a high volume of distribution, indicating good tissue penetration, together with a long terminal half-life for volasertib compared with BI 2536.^19^ Given these favorable PK properties that could potentially facilitate both intravenous (i.v.) and oral formulations, and promising preclinical efficacy and safety data,^19^ volasertib was prioritized for clinical development in both solid tumors and AML.

## Clinical development of volasertib in solid tumors

Clinical trials of volasertib in solid tumors are listed in Table 2. The first-in-man trial was a phase I, dose-escalation study in 65 patients with progressive advanced or metastatic solid tumors, who received a single 1 h infusion of volasertib every 3 weeks.^26^ The most commonly reported drug-related adverse events (AEs) were anemia (22% of patients), fatigue (15%), neutropenia (15%), thrombocytopenia (14%), nausea (9%), alopecia (9%) and febrile neutropenia (8%). Grade 3/4 AEs (24%) were mostly related to hematologic toxicity (18%), including dose-dependent neutropenia, both with and without infection, and thrombocytopenia. The main dose-limiting AE was neutropenia, and all cases of neutropenia and thrombocytopenia were reversible and manageable. The maximum-tolerated dose was established as 400 mg administered intravenously on day 1 every 3 weeks; however, 300 mg was selected for use in phase II trials owing to its generally manageable safety profile. PK analyses showed volasertib to have a large volume of distribution (>4000 l), moderate clearance (792 ml/min) and a long half-life (111 h).^26^ With no observed deviation from dose-linear PK behavior in the therapeutic relevant dose ranges, these data confirmed an improved PK profile of volasertib.^19^ The first-in-man trial also revealed signs of antitumor activity; three (5%) patients (urothelial cancer, ovarian cancer and melanoma) achieved partial responses (PRs) and 26 (40%) achieved stable disease, despite generally intense pretreatment (89% had received ⩾3 prior chemotherapies).^26^

A comparable phase I study in patients with solid tumors has been undertaken in 52 Asian patients, with results largely consistent with those of the first-in-man trial.^35^ The maximum-tolerated dose was identified as 300 mg when given on day 1 every 3 weeks, and 150 mg when administered on days 1 and 8 every 3 weeks. Dose-limiting toxicities were reversible thrombocytopenia, neutropenia and febrile neutropenia. Volasertib displayed some antitumor activity, with two (4%) patients (ureteral cancer and melanoma) achieving PR, while PK results showed a half-life of ~107 h and a large volume of distribution (4500 l).^35^ A separate phase I trial in Japanese patients with solid tumors is ongoing (Table 2).

A phase II trial of volasertib was undertaken in 50 patients with metastatic urothelial cancer following platinum failure.^36^ Although patients were heavily pretreated, volasertib demonstrated antitumor activity with seven (14%) patients achieving PR and 13 (26%) achieving stable disease as best response. The most common grade 3/4 AEs were neutropenia (28%), thrombocytopenia (20%) and anemia (16%); there was no evidence of cumulative toxicity.^36^

Two phase II trials of volasertib are ongoing in advanced ovarian cancer and advanced NSCLC (Table 2). Preliminary results from the ovarian cancer study indicate that single-agent volasertib shows antitumor activity comparable with that achieved with investigator's choice single-agent chemotherapy, while AEs were mainly hematologic and manageable.^37^ Initial data from the NSCLC study in patients with relapsed or refractory NSCLC after platinum-based first-line therapy show a lack of improvement in progression-free survival or overall response rate with combination volasertib and pemetrexed compared with single-agent pemetrexed.^38^

Although some patients with solid tumors have responded well to single-agent volasertib, with some patients able to achieve prolonged durations of response, overall antitumor activity has been modest in trials performed to date.^26,36^ Combination therapy is a potential option to improve efficacy, and volasertib has been combined with platinum chemotherapy to investigate this approach. In a phase I study in 61 heavily pretreated patients with advanced solid tumors, combination treatment was generally well tolerated, suggesting that volasertib is suitable for coadministration with cytotoxic chemotherapy at full single-agent doses.^39,40^ Five (8%) patients achieved PR, while 27 (44%) achieved stable disease or PR. Additional studies in advanced solid tumors are investigating volasertib combined with other agents, including trials involving the kinase inhibitors afatinib (NCT01206816) and nintedanib (NCT01022853), in which antitumor activity has been observed. Responses were observed in 2/29 patients treated with volasertib plus afatinib (PRs in both patients with NSCLC and head and neck cancer, respectively) and 2/30 patients treated with volasertib plus nintedanib (a complete response (CR) in a patient with breast cancer and a PR in a patient with NSCLC).^41,42^

## An unmet need for new therapies in AML

Treatment guidelines are well established for AML,^43, 44, 45^ a malignancy that can be challenging to manage in many cases.^46^ Induction chemotherapy with an anthracycline and cytarabine, as in the widely used ‘3+7' regimen, is standard; however, many patients with AML are of an older age and unable to tolerate this intensive treatment.^44,45^ Data from the past three decades, in which anthracyclines plus cytarabine was the most commonly used therapy for AML, demonstrate that little progress has been made in improving overall survival in patients aged ⩾60 years compared with those aged <60 years.^47,48^ In addition, there are various cytogenetic subtypes of AML that strongly influence response to treatment, and therapeutic resistance associated with adverse cytogenetics is common.^44,45^ Hypomethylating agents or low-dose cytarabine (LDAC) are options for some older, less-fit patients. Azacitidine has demonstrated a survival benefit vs conventional care regimens in older patients with AML and 20–30% blasts; however, no survival benefit has yet been determined with azacitidine in patients with AML and >30% blasts.^49^ Similarly, a randomized trial of LDAC vs palliative treatment in patients not considered eligible for intensive therapy demonstrated a better CR rate and improved survival with LDAC, but no benefit was seen in patients with adverse cytogenetics.^50^ In contrast, decitabine demonstrated improved CR rates, but primary analysis did not show a significant survival benefit compared with either supportive care or LDAC in older patients with newly diagnosed AML.^51^ More efficacious treatments are urgently needed,^46,52^ and as cell cycle inhibitors may be particularly useful in rapidly proliferating malignancies such as AML, there is a clear rationale for investigating Plk inhibitors in this setting.^8^ In this context, *in vitro* studies have shown that Plk1 is highly expressed in leukemic cell lines and tumor cell samples derived from patients with AML compared with normal hematopoietic progenitor cells.^7,53^ Furthermore, leukemic cells were shown to be more sensitive to Plk1 inhibition, as demonstrated by a marked decrease in cell proliferation, compared with normal progenitor cells.^7^

## Clinical development of volasertib in AML

The clinical development of volasertib in AML is well underway; reporting, ongoing and planned clinical trials are listed in Table 2. A phase I/II study evaluated the safety, efficacy and PKs of volasertib plus LDAC and volasertib monotherapy in patients with AML ineligible for intensive remission induction therapy.^54, 55, 56, 57^ This trial was performed in two parts: a phase I part and a phase IIa part. In the phase I part of the trial, volasertib was investigated in combination with LDAC (*n*=32) or as a monotherapy (*n*=56) in patients with relapsed/refractory AML to determine the maximum-tolerated dose. In the phase II part, the combination of volasertib plus LDAC (*n*=42) was compared with LDAC monotherapy (*n*=45) in patients with previously untreated AML to explore the efficacy of the combination schedule in comparison with LDAC monotherapy. The phase I, dose-escalation part of the trial, conducted in patients with relapsed/refractory AML, identified the recommended dose of volasertib as 450 mg every second week as a single agent and 350 mg every second week in combination with LDAC.^54, 55, 56^ At volasertib monotherapy doses ⩾350 mg, antileukemic activity was observed, with 5/43 (12%) patients achieving a complete remission with incomplete blood count recovery (CRi).^56^ In the volasertib plus LDAC combination arm, 7/32 patients achieved a CR or CRi.^55^ In the phase II part of the trial, preliminary data demonstrated a substantially higher objective response rate with volasertib (350 mg) plus LDAC vs LDAC alone with a CR/CRi rate of 31.0% vs 13.3% (*P*=0.052). Furthermore, subgroup analyses indicate that responses in the volasertib plus LDAC treatment arm occurred across all genetic groups (including adverse genetics). A CR was observed in 1/5 patients with *FLT3*-internal tandem duplication (ITD) and 3/7 patients with *nucleophosmin 1* mutations treated with volasertib plus LDAC.^57^ Median event-free survival (5.6 vs 2.3 months; *P*=0.021) and overall survival (8.0 vs 5.2 months; *P*=0.047) was also significantly improved for volasertib plus LDAC vs LDAC alone.^57^ While toxicity was increased with combination therapy, presumably owing to the myelosuppressive effect of volasertib, there was no evidence of increased early mortality for the combination regimen vs LDAC alone.^57^ PK analyses showed that volasertib exhibited multicompartmental behavior with moderate clearance, a large volume of distribution and a long terminal half-life,^55, 56, 57^ as seen in patients with advanced solid tumors.^26^

These encouraging data from the phase II part of this AML trial has prompted the initiation of a phase III trial of volasertib in AML (POLO-AML-2). This trial is designed to evaluate, in a randomized, double-blind setting, the efficacy and safety of volasertib plus LDAC vs placebo plus LDAC in 660 patients aged ⩾65 years with previously untreated AML who are ineligible for intensive remission induction therapy (NCT01721876). The primary end point of POLO-AML-2 is an objective response (CR and CRi), and the study is expected to be completed in early 2016 (final data collection date for primary outcome measure). A volasertib monotherapy, dose-escalation, phase I trial in Japanese patients with AML (NCT01662505) and another phase I study of volasertib plus decitabine, a hypomethylating agent approved by the European Medicines Agency for the treatment of patients ⩾65 years with AML, are ongoing (NCT02003573). Volasertib is also being investigated in a phase I trial in combination with azacitidine in patients with myelodysplastic syndromes or chronic myelomonocytic leukemia who are ineligible for high-intensity therapy (NCT01957644).

## Potential biomarkers of volasertib sensitivity

As discussed above, treatment with volasertib as a monotherapy and in combination with other agents resulted in significant clinical benefits, including prolonged durations of response, for subgroups of patients with solid tumors.^26,35, 36, 37,39, 40, 41, 42^ In AML, volasertib in combination with LDAC resulted in a CR/CRi rate of 31% of patients in a randomized, phase II trial of patients with previously untreated AML.^57^ As not all patients respond to volasertib therapy, predictive biomarkers are of interest to determine and/or predict drug activity and ultimately optimize therapy options for patients.

**Figure 5:**
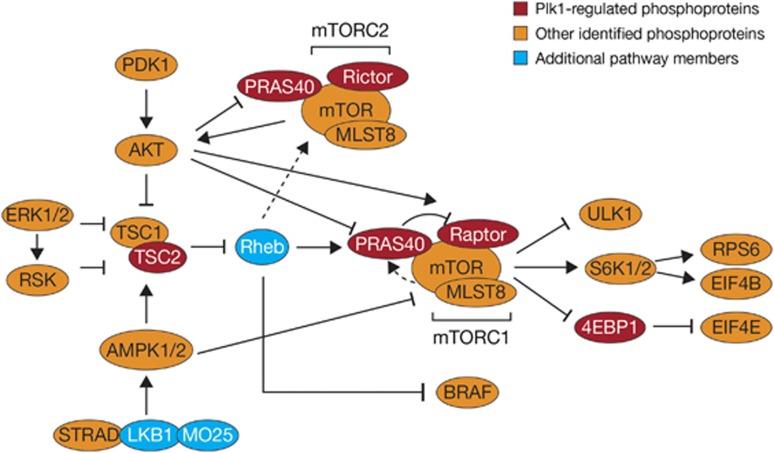
Example of non-mitotic functions of Plk1: effects on the mTOR pathway. Reproduced from Oppermann *et al.*^63^ with kind permission from the American Society for Biochemistry and Molecular Biology.

Proteomic analyses have identified proteins normally phosphorylated by Plk1, including many that are not involved with mitosis.^32^ These, and other proteins such as heat-shock protein 70,^58^ may be worth investigating as potential biomarkers of volasertib sensitivity. There is also a possibility that Plk1-regulated phosphoproteins may be implicated in serine/threonine kinase activation of the mammalian target of rapamycin (mTOR) pathway and a molecular and functional link between Plk1 and the mTOR pathway has been shown in AML.^59^ As the mTOR pathway displays aberrant regulation in hematologic malignancies such as AML,^60, 61, 62^ a further range of potential downstream markers could be investigated for sensitivity to volasertib (Figure 5).^32,63^ In this regard, it is noteworthy that a proteomics study in AML identified a signature of four phosphorylated proteins that act as predictors for response to the FLT3-targeted tyrosine kinase inhibitor, quizartinib.^64^ Proteomics of AML is still in its infancy,^65^ but may allow delineation of defined signal transduction and tumor suppressor pathways in single cells or cell extract assay:^66,67^ sample formats that may be relevant for future clinical trials of Plk1-targeting therapy. Relevant markers might be also identified based on comprehensive genomic, epigenomic and transcriptomic analyses.

Other possible biomarkers may include the breast cancer susceptibility protein, BRCA2, and the tumor suppressor protein, p53. Given that Plk1 has been shown to phosphorylate BRCA2,^68^ the presence or absence of this Plk1 substrate could potentially have some impact on volasertib susceptibility in solid tumors. In another study, p53 has been suggested as an *in vitro* predictive biomarker for the Plk1 inhibitor, GSK461364A, with the loss of p53 function resulting in sensitivity to the drug in a panel of human cancer cell lines.^69^ However, a separate study on colon cancer cell lines with volasertib showed no similar relationship.^70^ Clearly, the relationship between Plk1 and p53 is complex and further work is required to elucidate the full mechanism of their interaction in different malignancies.

Recently, volasertib has been reported to inhibit BRD4 binding at double-digit nanomolar concentrations in biochemical and binding assays; however, cellular effects on a BRD4 pharmacodynamic biomarker were only observed at concentrations of 300 nM.^27^ In published works, inhibitors of BRD4 appear more effective in *nucleophosmin 1* or *DNA (cytosine-5)-methyltransferase 3*-mutated AML cells,^71,72^ but a deeper understanding of the role of BRD4 as epigenetic modulator in AML is still missing. The concept of dual Plk1 and BRD4 inhibition may be interesting in light of ongoing trials with epigenetic modulators.^73^ The histone deacteylase inhibitor valproic acid has been shown to upregulate trimethylation of histone H3 lysine 27 in AML cells,^74^ and may be an interesting drug for volasertib combination therapy.

## Conclusions and future perspective

To date, the promising therapeutic effects of volasertib have been most clearly observed in patients with AML when given in combination with LDAC. In part, this may be due to the high mitotic index of the malignancy and its high expression of Plk1, combined with the balanced efficacy and safety profiles of this drug and its favorable PK characteristics. From a drug exposure perspective, the concentration of volasertib in the vicinity of malignant hematopoietic cells in the blood/bone marrow of AML patients may differ significantly from that in the tissue surrounding cancerous cells in solid tumors. Indeed, the malignant cells in AML reside in the same vascular system that delivers the drug into the body. Even small molecules like volasertib may have limited distribution in solid tumors owing to heterogeneous blood supply, elevated interstitial pressure and large transport distances in the interstitium,^75^ although this may be partially eliminated by the large distribution volume observed with volasertib.^26^ Finally, vigorous proliferation of malignant cells may be observed in AML, making this disease more susceptible to Plk1 inhibitors than solid tumors, whose cells tend to display a slower growth rate.

In the 25 years since intensive induction regimens became standard therapy for AML, little progress has been made in improving overall survival in patients aged ⩾60 years.^47,48^ An urgent need therefore exists for development of better therapies for AML, especially in older patients. The use of volasertib in combination therapy is a promising option requiring further exploration in this setting, particularly in previously untreated patients with AML who are ineligible for intensive induction therapy. Results of the POLO-AML-2 phase III AML trial will provide useful insights into the potential utility of this approach in this patient population.

## Figures and Tables

**Table 1:** Kinases not targeted by volasertib at concentrations up to 10 μmol/l

Abl	MAPKAP-K2
AMPK	MEK1
Aurora A	Met
Aurora B	MKK1
Axl	MSK1
B-RAF	Nek6
Btk	p38a
Cdk1/B1	p38b
Cdk2/E	p38d
CHK1	p38g
CK1	PAK2
CK2	PDGFRa
C-RAF	PDGFRb
CSK	PDK1
DYRK1A	PI3Ka
ECK	PKA
ErbB4	PKBa
ERK2	PKBb
FGFR1	PKCa
FGFR3	PRAK
FLT1	Ret
FLT3	ROCK2
GSK3β	Ron
Hek	S6K
HER2	SGK
HGFR	Src
ITK	βIRK
JAK2	Syk
JAK3	Tie2
JNK1	VEGFR1
Lck	VEGFR3
Lyn	

**Table 2:** Clinical trials of volasertib in solid and hematologic malignancies

*Description*	*Treatment*	*Dose*	*Indication*	*Number of patients*	*Reference*
*Trials in solid tumors*					
Phase I, dose escalation (NCT00969553)	Volasertib	12–450 mg	Advanced solid tumors	65	Schöffski *et al.*^26^
Phase I, dose escalation in Japanese patients (NCT01348347)	Volasertib	Schedule A (q3w): 100–300 mg; schedule B (q2w): 50–200 mg	Advanced solid tumors	52	Lin *et al.*^35^
Phase II, single arm, open label (NCT01023958)	Volasertib	300–350 mg	Locally advanced or metastatic urothelial cancer	50	Stadler *et al.*^36^
Phase II, randomized (NCT01121406)	Volasertib vs single-agent chemotherapy	Volasertib 300 mg or investigator's choice of paclitaxel, gemcitabine, topotecan or pegylated liposomal doxorubicin	Advanced ovarian cancer	109 Volasertib monotherapy (*n*=54)	Pujade-Lauraine *et al.*^37^
Phase II, open label, randomized (NCT00824408)	Volasertib monotherapy vs volasertib+pemetrexed vs pemetrexed monotherapy	Volasertib 300 mg or volasertib 300 mg plus pemetrexed 500 mg/m^2^, or pemetrexed 500 mg/m^2^	Advanced NSCLC	143 Volasertib monotherapy (*n*=37)	Ellis *et al.*^38^
Phase I, dose escalation (NCT00969761)	Volasertib+cisplatin or carboplatin	Volasertib 100–350 mg+cisplatin 60–100 mg/m^2^ or carboplatin AUC4–6	Advanced solid tumors	61	Deleporte *et al.*^39^ and Dumez *et al.*^40^
Phase I, dose escalation (NCT01206816)	Volasertib+afatinib	Volasertib 150–300 mg+afatinib 30–50 mg, days 2–21 (schedule A) or afatinib 50–90 mg, days 2–6 (schedule B)	Advanced solid tumors	57	Peeters *et al.*^41^
Phase I, dose escalation (NCT01022853)	Volasertib+nintedanib	Volasertib 100–450 mg+nintedanib 200 mg two times daily (except volasertib infusion day)	Advanced solid tumors	30	De Braud *et al.*^42^
					
*Trials in hematologic malignancies*					
Phase I/II, open label, randomized (NCT00804856)	Volasertib+LDAC vs volasertib monotherapy (phase I) or vs LDACmonotherapy (phase II)	Phase I: volasertib 150–500 mg vs volasertib 150–400 mg+LDAC 20 mg two times dailyPhase II: volasertib 350 mg+LDAC 20 mg two times daily vs LDAC 20 mg two times daily	AML	Phase I: 88 (volasertib monotherapy; *n*=56)	Bug *et al.*^54,55^ and Döhner *et al.*^56,57^
				Phase II: 87	
Phase III, double blind, randomized (NCT01721876; POLO-AML-2)	Volasertib+LDAC vs placebo+LDAC	NS	AML	660	Trial is onging; estimated completion date: April 2016^a^
Phase I, dose escalation in Japanese patients (NCT01662505)	Volasertib	NS	AML	20	Trial is onging; estimated completion date: July 2014^a^

Abbreviations: AML, acute myeloid leukemia; AUC, area under the curve; LDAC, low-dose cytarabine; NS, not stated; NSCLC, non-small-cell lung cancer; q2w, every 2 weeks; q3w, every 3 weeks.

a Final data collection date for primary outcome measure.

## Notes

BG has received compensation as a member of the scientific advisory board of Clavis Pharma AS (2012), BerGenBio AS and Boehringer Ingelheim GmbH and owns shares in Kinn Therapeutics AS and Alden Cancer Therapy AS. PS declares no conflict of interest.
